# Importance of depth and temperature variability as drivers of coral symbiont composition despite a mass bleaching event

**DOI:** 10.1038/s41598-023-35425-9

**Published:** 2023-06-02

**Authors:** Mariana Rocha de Souza, Carlo Caruso, Lupita Ruiz-Jones, Crawford Drury, Ruth D. Gates, Robert J. Toonen

**Affiliations:** 1 Hawai’i Institute of Marine Biology, School of Ocean and Earth Science and Technology, University of Hawai’i at Mānoa, Kāne’ohe, HI 96744 USA; 2 Chaminade University of Honolulu, 3140 Waialae Ave, Honolulu, HI 96816 USA

**Keywords:** Marine biology, Ecology

## Abstract

Coral reefs are iconic examples of climate change impacts because climate-induced heat stress causes the breakdown of the coral-algal symbiosis leading to a spectacular loss of color, termed ‘coral bleaching’. To examine the fine-scale dynamics of this process, we re-sampled 600 individually marked *Montipora capitata* colonies from across Kāne’ohe Bay, Hawai’i and compared the algal symbiont composition before and after the 2019 bleaching event. The relative proportion of the heat-tolerant symbiont *Durusdinium* in corals increased in most parts of the bay following the bleaching event. Despite this widespread increase in abundance of *Durusdinium*, the overall algal symbiont community composition was largely unchanged, and hydrodynamically defined regions of the bay retained their distinct pre-bleaching compositions. We explain ~ 21% of the total variation, of which depth and temperature variability were the most significant environmental drivers of Symbiodiniaceae community composition by site regardless of bleaching intensity or change in relative proportion of *Durusdinium*. We hypothesize that the plasticity of symbiont composition in corals may be constrained to adaptively match the long-term environmental conditions surrounding the holobiont, despite an individual coral’s stress and bleaching response.

## Introduction

Anthropogenic climate change impacts ecosystems across the globe^1^. Coral reefs are among the most iconic examples of climate-driven ecosystem decline, exhibiting a characteristic loss of color called ‘bleaching’ in response to thermal stress. Bleaching is the paling of corals resulting from the breakdown of the symbiosis between the cnidarian host and dinoflagellate algae of the family Symbiodiniaceae, which is responsible for meeting about 90% of reef-building coral energy requirement^2^. Because corals are metabolically dependent on this symbiosis, long periods in a bleaching state can deplete host energy supply and reserves^3–5^, impact coral growth^6,7^ reproduction^8–10^, and result in coral mortality^11–13^. The frequency of mass coral bleaching events worldwide has increased nearly fivefold in the past four decades^14–17^, resulting in significant losses of live coral in many parts of the world^18,19^. Despite visual recovery, the impacts of bleaching may persist for years^10,20^ and the increasing frequency and duration of marine heatwaves suggests there might not be enough time for corals to recover between bleaching events^18,21^. Ocean temperatures are predicted to rise 1–2 °C under best-case emission scenarios^22^, and coral persistence through increasingly frequent and severe heatwaves is dependent on the capacity to acclimatize or adapt to a rapidly changing environment^14,23–25^. These factors make coral reefs one of the most vulnerable ecosystems to increasing global temperatures^26,27^.

One mechanism by which corals may deal with thermal stress is through a change in the relative proportion of more thermally resistant algal endosymbionts hosted by corals that experience thermal stress^21,28^. Although some corals maintain stable associations or revert to pre-bleaching algal symbiont composition^29,30^, others change their algal symbiont community composition following bleaching events and can maintain altered proportions of algal symbionts after recovery from bleaching^31,32^. The association of coral with specific types of Symbiodiniaceae can directly influence how corals respond to environmental stress^33–36^. For example, corals dominated by Symbiodiniaceae from the genus *Durusdinium* (previously clade D^37^) tend to be more resilient to heat stress and thus experience less bleaching^38–40^, including in our study species, *Montipora capitata*^41,42^. Despite increasing resistance to bleaching, hosting the stress tolerant *Durusdinium* often comes at an energetic cost, decreasing the growth or metabolite exchange rate of the host, although a suite of both biotic and abiotic factors can modify such generalizations^41^.

While differential susceptibility to bleaching mortality among coral species is relatively well documented^12,43–45^, there is also ubiquitous intraspecific variation in coral bleaching^46–53^. Whereas temperature and irradiance are generally accepted to be the main environmental factors contributing to coral bleaching severity^18,54–57^, bleaching severity is often highly variable among individuals within and among nearby sites^41,44,58–60^. Bleaching severity can also be influenced by the environmental conditions the corals experienced before or during the heat stress^47,61–65^. Further, other environmental factors that can modify bleaching responses of corals often correlate with irradiance and temperature, such as depth^60,66^, sedimentation^67,68^, wave energy^69^, and flow^70^. Other factors such as suspended sediments^71,72^, nutrient input^73,74^, and acidification^75,76^ can exacerbate or ameliorate coral bleaching severity. Because these factors contribute to the breakdown of the coral-algal symbiosis, it seems likely that they may also impact the community composition of algal symbionts, but there is no strong consensus about the role of environmental drivers of spatial variation in coral algal symbiont community structure^77–81^.

Kāne’ohe Bay, the largest protected embayment in the Hawaiian Islands, has among the highest coral cover in Hawai’i^82,83^. The bay is environmentally and biologically heterogeneous^84–86^, with the northern extents experiencing higher circulation and lower mean residence times than the southern portion of the bay^87,88^. Interestingly, because Kāne’ohe Bay is relatively shallow and has high productivity and long residence times, the fluctuations in pCO_2_ and temperature are increased relative to open coastal reefs. Corals in Kāne’ohe Bay are exposed to temperature and acidification regimes that will not be seen for decades in other parts of the state^84^, leading to divergent environmental tolerances between the corals growing in Kāne’ohe Bay and conspecifics collected from exposed coastal reefs a few kilometers away^89^. Further, thermal tolerance experiments conducted in 2017 show that individuals take longer to bleach, maintain higher calcification rates, and experience lower bleaching mortality, than were observed for the same species at the same location in 1970^24^. Cumulatively, these results suggest corals in Kāne’ohe Bay have become more resistant to thermal stress, and may indicate an important role of environmental history in improving stress tolerance and susceptibility to coral bleaching.

The rice coral, *Montipora capitata*, is a dominant reef builder in Kāne’ohe Bay, where it hosts symbionts in the genera *Cladocopium*, *Durusdinium* or a mixed community^41,42,90–92^. The environmental heterogeneity of the bay and the complexity of symbiont communities in *M. capitata*, create an ideal system to investigate factors influencing coral bleaching response and resilience^84,89,93,94^. We previously quantified the algal symbiont across Kāne’ohe Bay in 2018^81^ before using this baseline to re-sample colonies after the 2019 bleaching event^95^ to compare algal symbiont community change through time. Here, we take advantage of this natural bleaching event to examine whether Symbiodiniaceae community structure changes in response to thermal stress, and if so, whether environmental factors modify the community response of algal symbionts within individual *M. capitata* colonies across the environmental mosaic of Kāne’ohe Bay.

## Results

### Symbiodiniaceae identification

In 2019, 496 colonies passed initial quality control steps, representing a loss of ~ 100 colonies due to missing tags, mortality, or other unknown causes. The relative representation of types, profiles and symbiont genera were broadly similar across years, representing the background distribution of Kāne’ohe Bay. A total of 214 Symbiodiniaceae types were identified in 2019, 178 (83%) in the genus *Cladocopium* and 36 (17%) in the genus *Durusdinium*. These numbers are similar to results from 2018, where 283 Symbiodiniaceae types were identified, 241 (85%) belonging to *Cladocopium*, and 42 (15%) belonging to the *Durusdinium*^81^. Twenty-nine ITS2 DIV profiles were identified across all samples in 2019, with twenty-five belonging to the genus *Cladocopium* and 4 belonging to the genus *Durusdinium*, consistent with 2018 when 26 ITS2 type profiles were identified across all samples, 23 of which were from the genus *Cladocopium*, with the remaining 3 belonging to the genus *Durusdinium*.

In 2019, 30% of colonies hosted *Durusdinium* only, an increase of 19% from 2018 when 11% hosted only *Durusdinium.* In 2019, 22% of colonies hosted a mixed community of both genera, 24% fewer than 2018, when 46% of colonies hosted a combination of both genera. In 2019, among mixed colonies (N = 109), 22 (20%) were dominated by *Cladocopium* (> 80% of reads identified as *Cladocopium*), and 38 (35%) were dominated by *Durusdinium* (> 80% of reads identified as *Durusdinium*), while the remaining 49 (45%) had moderate abundances of both genera.

### Symbiodiniaceae community composition before and after bleaching

**Figure 1:**
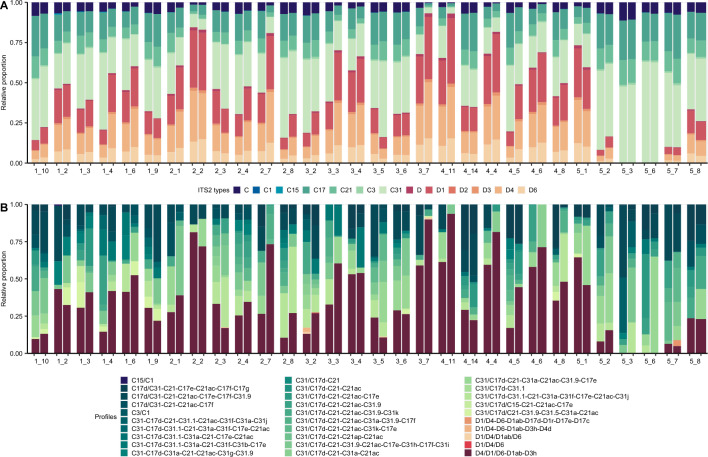
*Montipora capitata* Symbiodiniaceae community composition found in each of the 30 sites in Kāne’ohe Bay for (**A**) types and (**B**) profiles. A type refers to Symbiodiniaceae taxa that have a specific sequence as their most abundant sequence. A Symbiodiniaceae profile is a summary description set of ITS2 sequences that have been found in a sufficient number of samples (DIV). Here pairs of bars per site shows the algal symbiont community in 2018 and 2019, respectively. Symbiodiniaceae ITS2 subtypes were summarized to the major subtype to facilitate visualization in the bar charts (i.e., C31a and C31b were summarized as C31). Due to the wide diversity of ITS2 available in the SymPortal database, not all sequences are given names. Only DIV sequences are named, so unnamed *Cladocopium* and *Durusdinium* sequences were combined for visualization and represented as summed “C” and “D” types, respectively.

**Figure 2:**
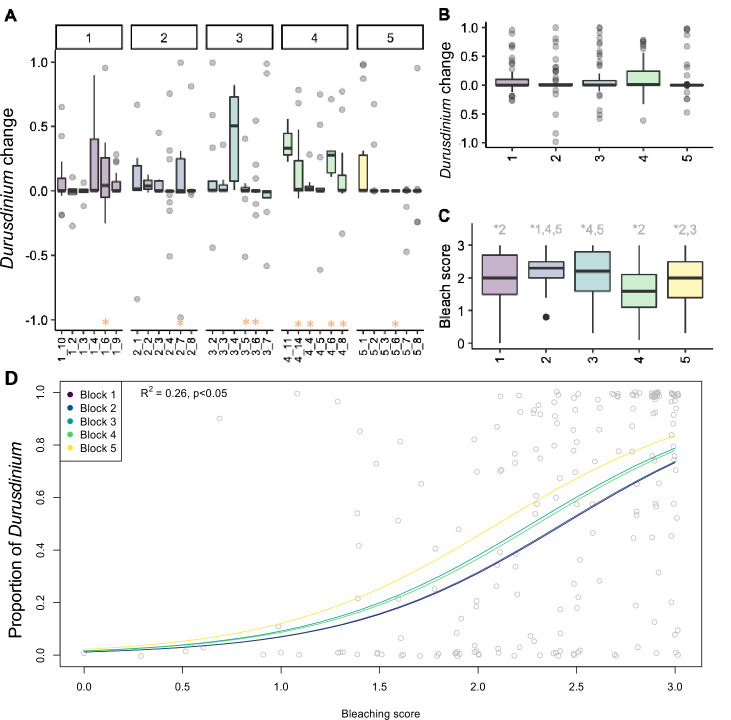
Change in *Durusdinium* in *M. capitata* in Kāne’ohe Bay in 2018 and 2019. (**A**) Boxplot of difference of proportion of *Durusdinium* in 2019 and 2018 in each site, in each of the 5 blocks. Sites in which the maximum temperature in 2019 exceeded + 1 °C above the previous year’s maximum are highlighted (*) on the axis. (**B**) Boxplot of difference of proportion of *Durusdinium* in 2019 and 2018 in each of the 5 blocks. (**C**) Bleaching score per block (3: healthy, 0: fully bleached); numbers at the top of (**C**) represent significant comparisons from pairwise PERMANOVA. (**D**) General linear regression of the proportion of *Durusdinium* per bleaching score, colored per block; McFadden’s R-square and significance included in the top left.

Corals at most sites had a combination of *Cladocopium* and *Durusdinium* regardless of year (Fig. 1, Supplemental material Fig. 1). Sites 5_3 and 5_6 in block 5 (the most northern part of the bay) were exceptions where we did not find *Durusdinium* in either year. In 2019, there was a general increase in the proportion of *Durusdinium* in sites located in all blocks following the bleaching event (Figs. 1, 2A); however, there was considerable variation between sites. For example, only site 5_1 in block 5 had an increased proportion of *Durusdinium*, while the other sites in block 5 remained nearly constant through time. Similarly, there was substantial variation in the change in proportion of *Durusdinium* from 2018 to 2019 in each block (Fig. 2B**,** Supplemental material Fig. 2A,B**)**, with block 4 presenting the highest increase, while block 2 and 5 had limited or no increases in *Durusdinium*. In each year, the proportion of *Durusdinium* hosted by colonies was lower in the northern and southern extremes of the bay (Fig. 1), which have relatively unique environments.

**Figure 3:**
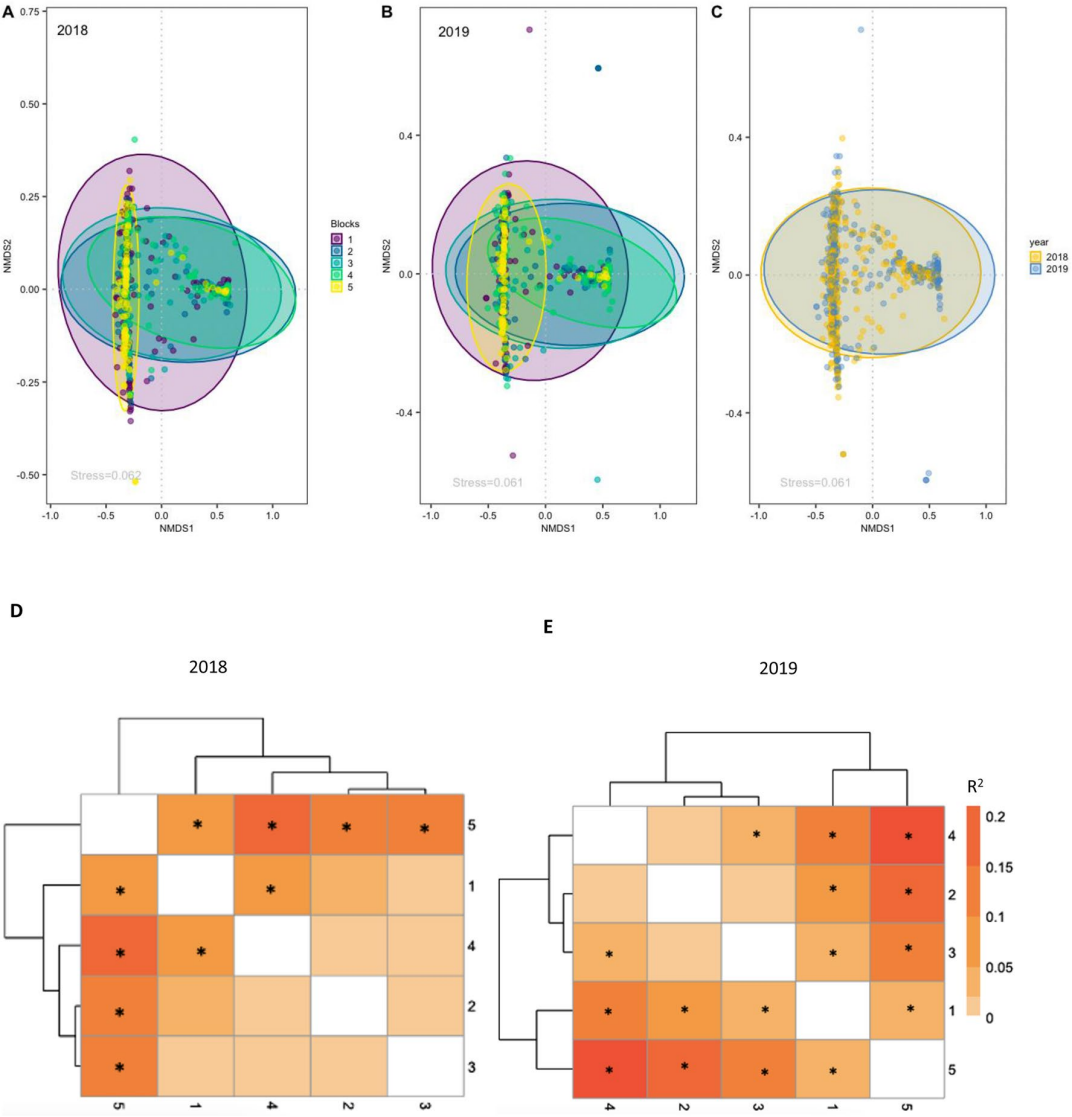
nMDS of Symbiodiniaceae types in *M. capitata* per regional block in Kāne’ohe Bay in (**A**) 2018 and (**B**) 2019 and (**C**) comparing both years. Heatmap of the R^2^ of the pairwise PERMANOVA of the Symbiodiniaceae diversity per block in 2018 (**D**) and in 2019 (**E**). Significant terms are marked with *.

Consistent with many other studies, bleaching stress resulted in a relative increase of *Durusdinium* among colonies of *M. capitata* across Kāne’ohe Bay in 2019 (Fig. 2A,B). Bleaching score (Fig. 2C**,** Supplemental material Fig. 2C) was significantly different between blocks, with the highest average score in block 4, and and lowest average score in block 2. Bleaching severity was negatively related to proportion of *Durusdinium* (Fig. 2D). Mean bleaching score in October 2019 for the 30 sites we surveyed in the bay was 2.2 (corresponding to paling), suggesting that *M. capitata* in the 2019 bleaching event experienced milder consequences compared to bay-wide estimates for previous bleaching events in which 62% (1996), 45% (2014) and 30% (2015) of colonies bleached^82^. Overall algal symbiont composition was significantly different between years (PERMANOVA F_9_ = 16.322, *p* = 0.001; Fig. 3C, Table 1).

**Table 1:** PERMANOVA based on Bray–Curtis dissimilarities of the *M. capitata* algal symbiont diversity present in corals sampled randomly from each environmentally defined block in Kāne’ohe Bay, considering 2018 and 2019 years, and 2019 only. Permanova results for 2018 only are present in De Souza et al.^81^.

Factor	Df	Sum Sq	F	R^2^	P
Year	1	0.206	3.763	0.003	0.038*
Year: Block	9	50.256	16.322	0.876	0.001*
2019 only					
Block	4	3.806	24.393	0.135	0.001*
Site: Block	25	6.134	0.245	6.290	0.001*
1 vs 2	1	0.745	13.617	0.067	0.001*
1 vs 3	1	0.372	7.122	0.039	0.005*
1 vs 4	1	1.201	24.091	0.109	0.001*
1 vs 5	1	0.276	6.845	0.033	0.003*
2 vs 3	1	0.062	1.064	0.006	0.315
2 vs 4	1	0.082	1.482	0.007	0.233
2 vs 5	1	2.045	43.999	0.172	0.001*
3 vs 4	1	0.218	4.089	0.021	0.031*
3 vs 5	1	1.290	29.622	0.132	0.001*
4 vs 5	1	2.844	67.115	0.235	0.001*

### Symbiodiniaceae spatial variation

There were significant differences in Symbiodiniaceae community composition among blocks of Kāne’ohe Bay observed after thermal stress in 2019 (PERMANOVA, F_25_ = 6.290, *p* = 0.001; Table 1). Although the large sample size of this study enables the detection of significant differences in overall community between years, the pairwise patterns between blocks remain consistent with results from 2018^81^. Blocks 1 and 5 were significantly different (Fig. 3D,E, Table 1) from each other and the center region of the bay, while blocks in the middle of the bay were largely indistinguishable (except for block 3 vs 4, Table 1). Compared to 2018, bleaching in 2019 intensified the differences among these two spatial groups^81^.

Despite a significant increase in the proportional representation of *Durusdinium* among the algal symbiont communities following bleaching, the underlying signal of geographic structure remained (Fig. 3A,B).

### Drivers of symbiont community composition

The 30 sites had broadly different environmental characteristics. Depth varied from 0.5 to 3.5 m; block 5 was the deepest block (mean 2.71 m), while sites in block 2 were shallowest (mean 1.36 m; Supplemental material Tables 1, 2). Sedimentation ranged nearly 300-fold from 0.01 to 2.93 g/day. In both years, sites in the middle of the bay had a smaller daily temperature range and daily temperature standard deviation when compared to sites in the northern and southern extremes of the bay (1 and 5). In 2019, block 4 had the highest increase in mean temperature and had the maximum absolute temperature (Supplemental material Tables 1, 2)**.**

**Figure 4:**
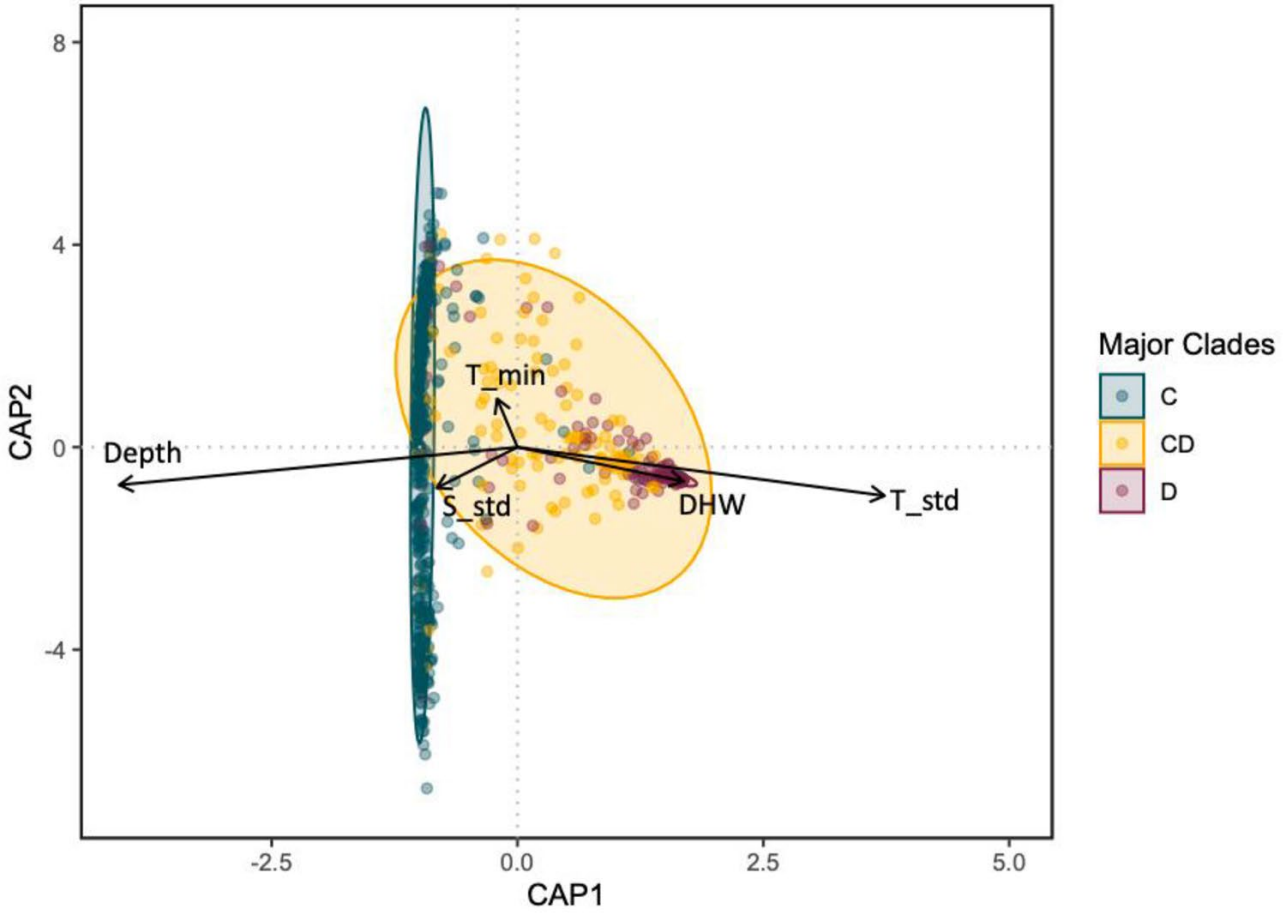
Distance based redundancy analysis (dbRDA) for environmental drivers of the Symbiodiniaceae communities measured in *Montipora capitata* in Kāne’ohe Bay after the 2019 bleaching event. Each point represents a *M. capitata* colony sampled irrespective of site. For visualization, samples were considered as majority *Cladocopium* (C) if they contain > 80%C, majority *Durusdinium* (D) if > 80% D, and mixed CD otherwise. Only vectors for the environmental factors contributing significantly to the algal symbiont diversity are plotted. Each arrow signifies the multiple partial correlation of the environmental driver in the RDA whose length and direction can be interpreted as indicative of its contribution to the explained variation. T_std (temperature daily standard deviation), DHW (degree heating weeks), T_min (minimum temperature), DHW (degree heating weeks), S_std (sedimentation standard deviation).

We used dbRDA to examine environmental drivers of Symbiodiniaceae community structure and found six factors were significant after multiple comparisons correction (p < 0.05; Fig. 4**, **Table 2). In order of decreasing variance explained, depth, mean daily standard deviation in temperature, minimum temperature, sedimentation standard deviation, degree heating weeks significantly impacted Symbiodiniaceae community. Interestingly, most of these factors, were determined to be the major environmental drivers of Symbiodiniaceae community composition prior to the bleaching event^81^. Consistent with many other studies, increase in *Durusdinium* after bleaching was higher in shallower sites (Supplemental material Fig. 3), which also correspond to sites with greater variation in temperature (mean daily temperature standard deviation).

**Table 2:** PERMANOVA of the environmental drivers of Bray–Curtis dissimilarities among Symbiodiniaceae communities in *M. capitata* among Kāne’ohe Bay sites. Relative contribution was calculated as the sum square of each environmental factor divided by the sum of all environmental sum squares. Environmental factors explain 21% of Symbiodiniaceae variation.

Environmental factors	Df	Sum Sq	F	P	Relative contribution
Depth (m)					
Nominal	1	17.585	156.348	0.001*	0.6246
Temperature (°C)					
DHW	1	0.382	3,967	0.040*	0.0329
Mean	1	0.171	1.520	0.211	0.0073
Maximum	1	0.164	1.459	0.197	0.0104
Minimum	1	1.475	13.110	0.001*	0.0116
Daily range	1	0.160	1.425	0.202	0.0205
Mean daily standard deviation	1	6.339	56.358	0.001*	0.1889
Sedimentation (g/day)					
Mean	1	0.059	0.522	0.553	0.0019
Maximum	1	0.139	1.231	0.267	0.0049
Minimum	1	0.239	2.123	0.126	0.0419
Mean daily standard deviation	1	1.008	8.958	0.001*	0.0006
Total		27.721			
Residual		99.878			

## Discussion

Temperature and irradiance are generally the main environmental factors underlying breakdown in the symbiosis between the coral host and their endosymbiotic community which drives coral bleaching^18,54–57^. However, intraspecific variability in bleaching severity within and among sites is also well documented^41,44,58–60^, including in our study system^20,41,52^. The extent to which endosymbiotic Symbiodiniaceae community composition contributes to such bleaching variability remains uncertain: some corals maintain stable associations throughout bleaching or revert to pre-bleaching algal symbiont composition^29,30^, whereas others maintain an altered algal symbiont community composition following bleaching events^31,32^. While there has been considerable research examining coral algal symbiont communities, the role of environmental factors in contributing to variation in both algal symbiont community structure and coral bleaching responses remains comparatively understudied^46,50,52,96–98^.

We previously quantified the algal symbiont community of *M. capitata* in relation to environmental gradients throughout Kāne’ohe Bay in 2018^81^. Here, we resampled the same colonies following a natural bleaching event to examine Symbiodiniaceae diversity across an environmental mosaic and evaluate the relative importance of acute and chronic environmental conditions on symbiont communities. Like previous studies that often report an increase in heat-tolerant algal symbiont lineages when exposed to stressful conditions^34,77,99–102^, we found a significant increase in *Durusdinium* when comparing Symbiodiniaceae communities of individually marked *M. capitata* colonies sampled before (early 2018) and shortly after (October 2019) the bleaching event. In addition, we show that the proportion of *Durusdinium* in a coral is negatively correlated with bleaching severity, supporting the potential for positive fitness consequences after bleaching if corals acquire thermally tolerant symbionts, consistent with the adaptive bleaching hypothesis^103^. Cunning et al.^100^ found that at intermediate or low stress, corals decrease their proportion of heat stress algal symbionts (*Durusdinium*), while at higher stress (severe bleaching), the proportion of *Durusdinium* increases. Similarly, we found that block 4 had the highest maximum temperature increase, the most bleaching, and showed the greatest increase in *Durusdinium* compared to colonies in the other blocks.

Consistent with our previous study, the coral algal symbiont association was strongly influenced by environmental gradients. *Montipora capitata* located at the extreme southern and northern portions of Kāne’ohe Bay hosted Symbiodiniaceae communities that were significantly different from the center of the bay and may be reflective of the unique hydrodynamic regimes and environments in these regions. The environmental drivers included in the study explained only ~ 21% of the symbiont variation, but this is still considerably higher than the average for ecological and evolutionary studies^104^. Thus, while our findings highlight the importance of these environmental factors, about 80% of the variation remain unexplained, indicating that additional factors should be considered to fully understand the ecological dynamics of Symbiodiniaceae populations in the Bay. Future studies including water residency, wave action, nutrients, light, and coral host genotype, for example, are likely to provide additional insight into the primary drivers of coral symbiont community composition.

Despite a significant increase in the overall proportion of *Durusdinium* following the coral bleaching event, the same factors measured in 2018 (depth, variability in temperature and sedimentation) emerge as primary drivers of Symbiodiniaceae community composition among the factors we measured. In fact, 2018 and 2019 occupy almost the same multidimensional scaling space regardless of the bleaching event (Fig. 3). Corals dominated by *Durusdinium* are much more similar (closer together within the distance-based redundancy analysis) than those dominated by *Cladocopium* or with a mixed algal symbiont community composition, which corresponds to the reduced diversity of *Durusdinium* types and profiles we observed. Overall, these results suggest that the algal symbiont community composition was altered due to bleaching stress, but that such change was relatively minor in comparison to the previously established differences in community structure observed across the consistent environmental gradient of Kāne’ohe Bay. Interestingly, a similar pattern is reported by Botté et al.^105^ who found that reef location, rather than severity of bleaching, had the greatest impact on the microbiome of *Pocillora acuta* along the Great Barrier Reef. Based on these results, we hypothesize that the plasticity of symbiont composition in corals may be constrained to adaptively match the long-term environmental conditions surrounding the holobiont, despite the individual bleaching responses of corals in response to thermal stress.

Our result is concordant with Dilworth et al.^42^, who found consistency among the sites in the relative proportion of *Cladocopium* and *Durusdinium* in Kāne’ohe Bay. Interestingly, Dilworth et al.^42^, following a short thermal stress, reported a loss of *Cladocopium* in the mixed colonies, and higher recovery of *Durusdinium* compared to *Cladocopium* in bleached colonies. This finding may suggest that the algal symbiont community composition was starting to change but was not significant within the short time frame of the experiment. Alternatively, not all colonies may show such changes and this variability among individuals could make it difficult to detect significant changes. Cunning et al.^41^ surveyed 60 colonies of *M. capitata* 6 months after the 2014 mass bleaching event and found that the dominant algal symbiont genus remained the same for 80% of colonies throughout their study. The remaining 20% showed variability in the dominant algal symbiont genus through time, but the changes that occurred were in either direction (i.e., both C to D and D to C) and were not related to visual bleaching. However, with relatively few colonies sampled, and less than 20% showing a change in dominant algal symbiont type, there is relatively little power in these previous studies to determine significance of directionality. In the hundreds of colonies sampled here, we show a slight but significant overall increase in the proportion of *Durusdinium* following the bleaching event. Consistent with these previous studies, the identity of the coral host and the local environmental history both appear to be important drivers of algal symbiont community composition, because despite a slight general increase in the proportion of *Durusdinium*, there remains a strong and essentially unchanged signature of the original environmental gradient on algal symbiont community from prior to the bleaching event^81^. *M. capitata* is a vertical transmitter that releases symbiont provisioned eggs^106^, creating a tight co-evolutionary linkage between host and symbionts. Host genetic differentiation in functional ontologies is also strongly associated with symbiont community^92^, creating the potential for an environmental-symbiont linkage mediated by local adaptation of the host or holobiont.

While the increase in temperature during 2019 is an acute stress that led to bleaching, corals in different parts of the bay are also exposed to long-term environmental conditions that can act as chronic stressors, and might explain the mosaic spatial pattern of bleaching and Symbiodiniaceae composition across Kāne’ohe Bay. For example, in other studies, corals with a long history of exposure to variable microhabitats were more heat tolerant than nearby conspecifics sampled from more stable regimes^36,107–112^. This resilience imparted by more variable environments may result from an “ecological memory”^113–116^ that can play a significant role in determining how individual corals will respond to a given stress. Environmental memory in corals following consecutive events has been documented in a few studies (e.g.^82,120,121^). Although most mechanisms of environmental memory may be driven by the coral host^51^, a change in algal symbiont composition (either shuffling of relative proportions or shifting to novel symbionts) may also play an important role. However, the relevance of such environmental memory remains controversial, with some studies suggesting a short duration, as corals revert to their initial algal symbiont compositions^102^, whereas others report transgenerational inheritance of shuffled symbionts and suggest such change has major ecological relevance^117^. It is interesting to note that corals in site 5_3 and 5_6 in block 5 did not host *Durusdinium* in any of the years. Possible explanations for this pattern include (1) *Durusdinium* is not available for the corals in those sites, (2) environmental drivers favor the selection of *Cladocopium* versus *Durusdinium* in those sites, or (3) the algal symbiont composition in those locations is driven by the host coral genetics, where local adaptation creates a tradeoff to hosting this genus. While Caruso and De Souza et al.^86^ did not find a pattern of clonality in Kāne’ohe Bay, when surveying the same colonies, it remains unclear what genetic signals in the host would drive the Symbiodiniaceae composition. Taken together with our findings here, these studies indicate that Symbiodiniacae community composition responds to some combination of acute and chronic stressors, and that a better understanding of differences among host and algal symbiont species as well as drivers of environmental memory will improve our ability to predict coral bleaching at the level of individual colonies.

## Conclusions

Resampling of individually marked *M. capitata* colonies across the environmental mosaic of Kāne’ohe Bay following a natural bleaching event revealed that patterns of algal symbiont distribution change as predicted. A marine heatwave resulted in a significant increase in the proportion of *Durusdinium* detected overall. Among the variables measured in the study however, there is strikingly little change in either the primary environmental drivers of Symbiodiniaceae community structure (depth and temperature variability), nor the relative magnitude of those drivers in a distance-based redundancy analysis following the bleaching. Additionally, the nMDS of Symbiodiniaceae community composition is virtually indistinguishable following the bleaching from the previous year. Among the variables we measured, the primary drivers of Symbiodiniaceae community structure remained consistently associated with depth and daily temperature variability despite the increased representation by *Durusdinium* in response to the bleaching event. We hypothesize that this consistency in algal symbiont communities across chronic environmental gradients results from environmental memory such that while communities may respond to short-term acute stressors such as heat waves, they appear constrained by the long-term environmental conditions surrounding the holobiont.

## Material and methods

### Site selection and tagging

**Figure 5:**
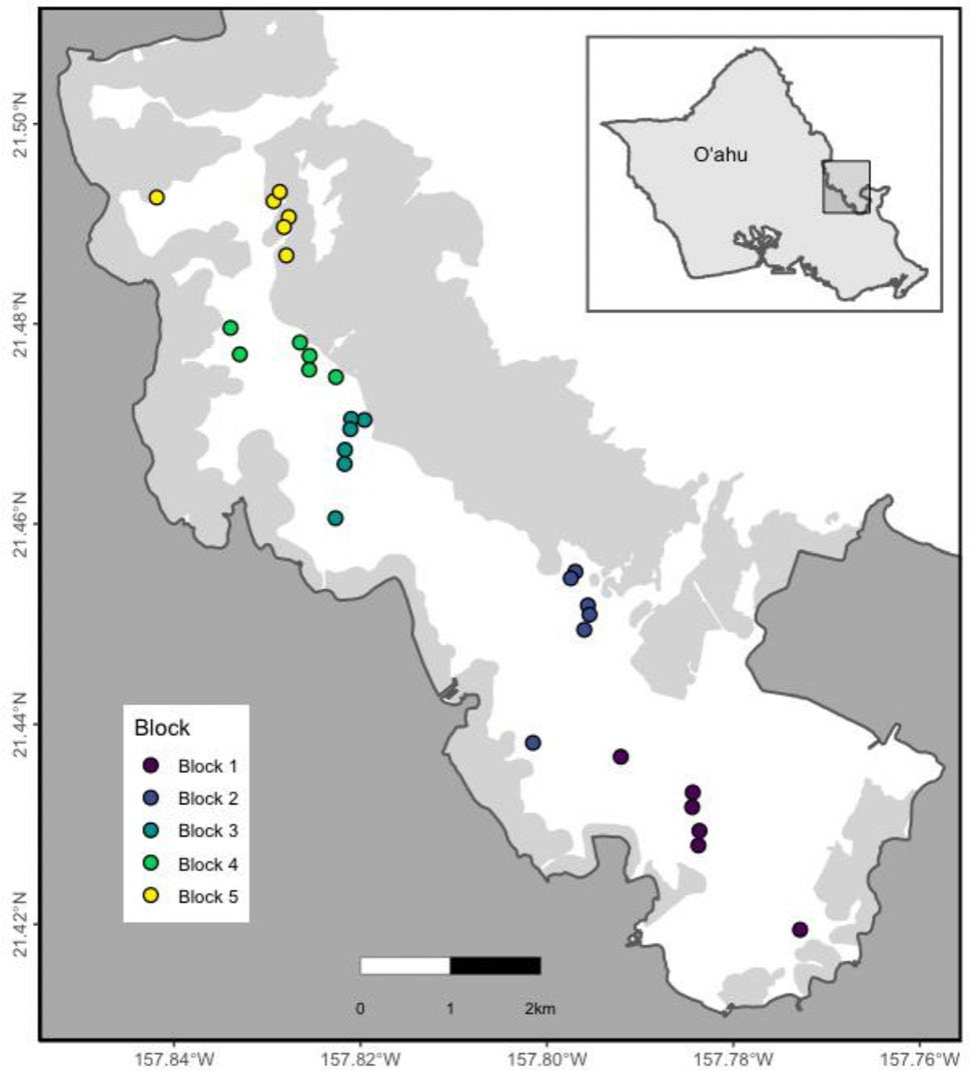
Map of Kāne’ohe Bay highlighting the location of each of the 30 sites across the bay.

*Montipora capitata* colonies were tagged on 30 patch reefs in Kāne’ohe Bay, O’ahu, Hawai’i, under SAP permit 2018-03 and SAP 2019-16 to HIMB from Hawai’i Department of Aquatic Resources. Kāne’ohe Bay was divided into 5 ‘blocks’ based on modeled water flow regimes and water residence times^88^ and six sites^86^ were selected in each block using stratified random sampling within habitats designated as patch reefs (Fig. 5). Site IDs consist of the digit corresponding to the block in which the site is contained, followed by the site number (e.g., 1_10, with six sites per block, but not necessarily in consecutive order).

Temperature loggers (Hobo Pendant from Onset Computer Corp: UA-001-64 Data Logger) were deployed at the center of each site. Temperature recordings every 10 min began on 12 July 2017 and continued until 26 July 2019, with the loggers periodically retrieved and recalibrated throughout the study period. Sediment traps were also deployed at the center of the block and exchanged every 1–2 months, and the weight of sediment was used to estimate the sedimentation rate in each site following^118^. See Refs.^81,86^ for details on site selection, host genetic sampling, and environmental data collection and analysis.

Twenty *M. capitata* colonies were tagged at each site and 1 cm^2^ clippings of each colony were collected in 2018 from visually healthy colonies. During the 2019 bleaching event, the colonies were re-visited between 3 and 21 October 2019, photographed and recollected. Sampled fragments were immediately preserved in 70% ethanol and stored at − 20 °C until processed. DNA from colonies collected in both years was extracted using the Nucleospin Tissue Kits (Macherey–Nagel, Düren, Germany) following manufacturer’s instructions.

During field sampling in October 2019, corals were assigned a visual bleaching score^24,52,93,119^: (0) totally bleached (> 80% of colony white with no visible pigmentation); (1) pale (60–80% colony affected by pigment loss); (2) pale (10–50% colony affected by pigment loss); (3) fully pigmented (< 10% colony with any visible paling)^52^. Each colony was scored two times independently by two different observers using in situ photographs taken during collection and the mean value was assigned as the bleaching score.

### Symbiodiniaceae ITS2 amplicon sequencing library preparation

ITS2 amplicon libraries were prepared from extracted DNA and sequenced following De Souza et al.^120^. Briefly, the ITS2 region was amplified for each sample, pooled and sequenced on an Illumina MiSeq platform (v3 2 × 300 bp PE) at University of Hawai’i at Manoa. Raw sequences were demultiplexed and quality filtered using Cutadapt^121^. To ensure differences in read number did not impact results or interpretation, we excluded 20 samples from 2018 and 24 from 2019 whose number of reads were more than 2 standard deviations above or below the mean. Forward and reverse reads were submitted to SymPortal^122^, a platform for identifying Symbiodiniaceae using high throughput ITS2 sequence data that differentiates intra‐ and intergenomic sources of ITS2 sequence variance. Sets of ITS2 sequences, occurring in a sufficient number of samples within both the dataset being analyzed and the entire database of samples run through SymPortal were identified as ‘defining intragenomic variants’ (DIVs) which were then used to characterize ITS2 type profiles.

In this study, we analyzed data based on Symportal outputs for Symbiodiniaceae “type” and “profile”. A type refers to Symbiodiniaceae taxa that have a specific sequence as their most abundant sequence. A Symbiodiniaceae profile is a summary description set of ITS2 sequences that have been found co-occurring in a sufficient number of samples (DIV).

### Statistical analysis

All analyses and figures were completed in R 2021.09.0 + 351 version (R Core Team, 2020). Map in Fig. 5 was done using *sf* and *ggplot2* packages. We calculated a variety of summary statistics from the temperature time series for each site^86^: mean daily temperature, average daily range, mean daily standard deviation, global mean, maximum and minimum temperature at each site. We calculated Degree heating weeks (DHW) per site as the accumulated time when temperature was above the bleaching threshold, set as 28.5 °C given a MMM of 27.5 °C^42,123^.

To examine if the relative proportion of heat resistant algal symbionts changed following a bleaching event, we calculated the relative proportion of *Durusdinium* in each colony from relative abundance values in 2018 and 2019^81^ and analyzed these data by site and block.

We used non-metric multidimensional scaling (NMDS) and permutational analysis of variance (PERMANOVA) in the R package *vegan* to examine symbiont community differentiation (Bray–Curtis dissimilarities) by year, block and site nested within block. We used the function pairwise.adonis to compare each block in 2018 and in 2019 and visualized the R^2^ from the PERMANOVA in a dendrogram using the package *pheatmap.*

To examine the influence of environmental factors on Symbiodiniaceae composition, we used a distance-based redundancy analysis (dbRDA) based on Bray–Curtis distances and tested the significance of each environmental driver using *vegan.* We calculated variance explained by each environmental variable as a proportion of total variance explained by environment (i.e., excluding residuals). We designated colonies with a relative abundance of > 80% from a single genus as majority *Cladocopium* (C) or *Durusdinium* (D), with all remaining samples designated as mixed CD, corresponding to corals with no dominant algal symbiont genus.

## Supplementary Information

Supplementary Information.

## Data Availability

The datasets generated used in the current study are available in the GitHub repository https://github.com/MarianaRochadeSouza/Symb-KBay-2018-2019.

## References

[1] Parmesan C (2006). Ecological and evolutionary responses to recent climate change. Annu. Rev. Ecol. Evol. Syst..

[2] Muscatine L, Porter JW (1977). Reef corals: Mutualistic symbioses adapted to nutrient-poor environments. Bioscience.

[3] Grottoli AG, Rodrigues LJ, Juarez C (2004). Lipids and stable carbon isotopes in two species of Hawaiian corals, *Porites compressa* and *Montipora verrucosa*, following a bleaching event. Mar. Biol..

[4] Rodrigues LJ, Grottoli AG (2007). Energy reserves and metabolism as indicators of coral recovery from bleaching. Limnol. Oceanogr..

[5] Wall CB, Ritson-Williams R, Popp BN, Gates RD (2019). Spatial variation in the biochemical and isotopic composition of corals during bleaching and recovery. Limnol. Oceanogr..

[6] Cantin NE, Lough JM (2014). Surviving Coral bleaching events: *Porites* growth anomalies on the Great Barrier Reef. PLoS ONE.

[7] Gold Z, Palumbi SR (2018). Long-term growth rates and effects of bleaching in *Acropora hyacinthus*. Coral Reefs.

[8] Baird A, Marshall P (2002). Mortality, growth and reproduction in scleractinian corals following bleaching on the Great Barrier Reef. Mar. Ecol. Prog. Ser..

[9] Fisch J, Drury C, Towle EK, Winter RN, Miller MW (2019). Physiological and reproductive repercussions of consecutive summer bleaching events of the threatened Caribbean coral *Orbicella faveolata*. Coral Reefs.

[10] Johnston EC, Counsell CWW, Sale TL, Burgess SC, Toonen RJ (2020). The legacy of stress: Coral bleaching impacts reproduction years later. Funct. Ecol..

[11] Depczynski M (2013). Bleaching, coral mortality and subsequent survivorship on a West Australian fringing reef. Coral Reefs.

[12] Loya Y (2001). Coral bleaching: The winners and the losers. Ecol. Lett..

[13] Magel JMT, Burns JHR, Gates RD, Baum JK (2019). Effects of bleaching-associated mass coral mortality on reef structural complexity across a gradient of local disturbance. Sci. Rep..

[14] Donner SD, Skirving WJ, Little CM, Oppenheimer M, Hoegh-Guldberg O (2005). Global assessment of coral bleaching and required rates of adaptation under climate change. Glob. Change Biol..

[15] Hoegh-Guldberg O (1999). Climate change, coral bleaching and the future of the world’s coral reefs. Mar. Freshwater Res..

[16] Oliver JK, Berkelmans R, Eakin CM, van Oppen MJH, Lough JM (2018). Coral bleaching in space and time. Coral Bleaching: Patterns, Processes, Causes and Consequences.

[17] Sully S, Burkepile DE, Donovan MK, Hodgson G, van Woesik R (2019). A global analysis of coral bleaching over the past two decades. Nat. Commun..

[18] Hughes TP (2017). Global warming and recurrent mass bleaching of corals. Nature.

[19] Stuart-Smith RD, Brown CJ, Ceccarelli DM, Edgar GJ (2018). Ecosystem restructuring along the Great Barrier Reef following mass coral bleaching. Nature.

[20] Wall CB (2021). Shifting baselines: Physiological legacies contribute to the response of reef corals to frequent heatwaves. Funct. Ecol..

[21] Claar DC (2020). Dynamic symbioses reveal pathways to coral survival through prolonged heatwaves. Nat. Commun..

[22] Pörtner, H. O. *et al.* IPCC, 2022: Summary for Policymakers.

[23] Bay LK, Doyle J, Logan M, Berkelmans R (2016). Recovery from bleaching is mediated by threshold densities of background thermo-tolerant symbiont types in a reef-building coral. R. Soc. Open Sci..

[24] Coles SL (2018). Evidence of acclimatization or adaptation in Hawaiian corals to higher ocean temperatures. PeerJ.

[25] Logan CA, Dunne JP, Eakin CM, Donner SD (2014). Incorporating adaptive responses into future projections of coral bleaching. Glob. Change Biol..

[26] Carpenter KE (2008). One-third of reef-building corals face elevated extinction risk from climate change and local impacts. Science.

[27] Hoegh-Guldberg O, Poloczanska ES, Skirving W, Dove S (2017). Coral reef ecosystems under climate change and ocean acidification. Front. Mar. Sci..

[28] Silverstein RN, Cunning R, Baker AC (2015). Change in algal symbiont communities after bleaching, not prior heat exposure, increases heat tolerance of reef corals. Glob. Change Biol..

[29] Smith EG, Vaughan GO, Ketchum RN, McParland D, Burt JA (2017). Symbiont community stability through severe coral bleaching in a thermally extreme lagoon. Sci. Rep..

[30] Thornhill DJ, LaJeunesse TC, Kemp DW, Fitt WK, Schmidt GW (2006). Multi-year, seasonal genotypic surveys of coral-algal symbioses reveal prevalent stability or post-bleaching reversion. Mar. Biol..

[31] Jones AM, Berkelmans R, van Oppen MJH, Mieog JC, Sinclair W (2008). A community change in the algal endosymbionts of a scleractinian coral following a natural bleaching event: Field evidence of acclimatization. Proc. R. Soc. B..

[32] Silverstein RN, Cunning R, Baker AC (2017). Tenacious D: *Symbiodinium*in clade D remain in reef corals at both high and low temperature extremes despite impairment. J. Exp. Biol..

[33] Abrego D, Ulstrup KE, Willis BL, van Oppen MJH (2008). Species–specific interactions between algal endosymbionts and coral hosts define their bleaching response to heat and light stress. Proc. R. Soc. B..

[34] Berkelmans R, van Oppen MJH (2006). The role of zooxanthellae in the thermal tolerance of corals: A ‘nugget of hope’ for coral reefs in an era of climate change. Proc. R. Soc. B..

[35] Howells EJ (2012). Coral thermal tolerance shaped by local adaptation of photosymbionts. Nat. Clim. Change.

[36] Oliver TA, Palumbi SR (2011). Do fluctuating temperature environments elevate coral thermal tolerance?. Coral Reefs.

[37] LaJeunesse TC (2018). Systematic revision of Symbiodiniaceae highlights the antiquity and diversity of coral endosymbionts. Curr. Biol..

[38] Rowan R (2004). Thermal adaptation in reef coral symbionts. Nature.

[39] Stat M, Gates RD (2011). Clade D *Symbiodinium* in Scleractinian corals: A “nugget” of hope, a selfish opportunist, an ominous sign, or all of the above?. J. Mar. Biol..

[40] Ulstrup K, Berkelmans R, Ralph P, van Oppen M (2006). Variation in bleaching sensitivity of two coral species across a latitudinal gradient on the Great Barrier Reef: The role of zooxanthellae. Mar. Ecol. Prog. Ser..

[41] Cunning R, Ritson-Williams R, Gates R (2016). Patterns of bleaching and recovery of *Montipora capitata* in Kāne‘ohe Bay, Hawai‘I, USA. Mar. Ecol. Prog. Ser..

[42] Dilworth J, Caruso C, Kahkejian VA, Baker AC, Drury C (2021). Host genotype and stable differences in algal symbiont communities explain patterns of thermal stress response of *Montipora capitata* following thermal pre-exposure and across multiple bleaching events. Coral Reefs.

[43] Adjeroud M (2009). Recurrent disturbances, recovery trajectories, and resilience of coral assemblages on a South-Central Pacific reef. Coral Reefs.

[44] Marshall PA, Baird AH (2000). Bleaching of corals on the Great Barrier Reef: Differential susceptibilities among taxa. Coral Reefs.

[45] van Woesik R, Sakai K, Ganase A, Loya Y (2011). Revisiting the winners and the losers a decade after coral bleaching. Mar. Ecol. Prog. Ser..

[46] Wagner D, Kramer P, van Woesik R (2010). Species composition, habitat, and water quality influence coral bleaching in southern Florida. Mar. Ecol. Prog. Ser..

[47] Guest JR (2012). Contrasting patterns of coral bleaching susceptibility in 2010 suggest an adaptive response to thermal stress. PLoS ONE.

[48] Pratchett MS, McCowan D, Maynard JA, Heron SF (2013). Changes in bleaching susceptibility among corals subject to ocean warming and recurrent bleaching in Moorea, French Polynesia. PLoS ONE.

[49] Grottoli AG (2014). The cumulative impact of annual coral bleaching can turn some coral species winners into losers. Glob. Change Biol..

[50] Hoogenboom MO (2017). Environmental drivers of variation in bleaching severity of acropora species during an extreme thermal anomaly. Front. Mar. Sci..

[51] Drury C (2020). Resilience in reef-building corals: The ecological and evolutionary importance of the host response to thermal stress. Mol. Ecol..

[52] Matsuda SB (2020). Coral bleaching susceptibility is predictive of subsequent mortality within but not between coral species. Front. Ecol. Evol..

[53] Drury C, Lirman D (2021). Genotype by environment interactions in coral bleaching. Proc. R. Soc. B..

[54] Bahr KD, Jokiel PL, Rodgers KS (2016). Influence of solar irradiance on underwater temperature recorded by temperature loggers on coral reefs: Evaluation of underwater temperature loggers. Limnol. Oceanogr. Methods.

[55] Berkelmans R, De’ath G, Kininmonth S, Skirving WJ (2004). A comparison of the 1998 and 2002 coral bleaching events on the Great Barrier Reef: Spatial correlation, patterns, and predictions. Coral Reefs.

[56] Brown BE (1997). Coral bleaching: Causes and consequences. Coral Reefs.

[57] Glynn PW (1996). Coral reef bleaching: Facts, hypotheses and implications. Glob. Change Biol..

[58] Bruno J, Siddon C, Witman J, Colin P, Toscano ME (2001). Niño related coral bleaching in Palau, Western Caroline Islands. Coral Reefs.

[59] Green RH, Lowe RJ, Buckley ML, Foster T, Gilmour JP (2019). Physical mechanisms influencing localized patterns of temperature variability and coral bleaching within a system of reef atolls. Coral Reefs.

[60] Penin L, Adjeroud M, Schrimm M, Lenihan HS (2007). High spatial variability in coral bleaching around Moorea (French Polynesia): Patterns across locations and water depths. C. R. Biol..

[61] Dunne R, Brown B (2001). The influence of solar radiation on bleaching of shallow water reef corals in the Andaman Sea, 1993–1998. Coral Reefs.

[62] Brown B, Dunne R, Goodson M, Douglas A (2002). Experience shapes the susceptibility of a reef coral to bleaching. Coral Reefs.

[63] Jokiel PL, Brown EK (2004). Global warming, regional trends and inshore environmental conditions influence coral bleaching in Hawai‘i : Coral bleaching in Hawai‘i. Glob. Change Biol..

[64] Bellantuono AJ, Hoegh-Guldberg O, Rodriguez-Lanetty M (2012). Resistance to thermal stress in corals without changes in symbiont composition. Proc. R. Soc. B..

[65] Ainsworth TD (2016). Climate change disables coral bleaching protection on the Great Barrier Reef. Science.

[66] Muir PR, Marshall PA, Abdulla A, Aguirre JD (2017). Species identity and depth predict bleaching severity in reef-building corals: Shall the deep inherit the reef?. Proc. R. Soc. B..

[67] Anthony KRN, Connolly SR, Hoegh-Guldberg O (2007). Bleaching, energetics, and coral mortality risk: Effects of temperature, light, and sediment regime. Limnol. Oceanogr..

[68] Duckworth A, Giofre N, Jones R (2017). Coral morphology and sedimentation. Mar. Pollut. Bull..

[69] Obura, D., Grimsditch, G., & International Union for Conservation of Nature and Natural Resources. Resilience assessment of coral reefs: Assessment protocol for coral reefs, focusing on coral bleaching and thermal stress. (IUCN, 2009).

[70] Nakamura T, van Woesik R (2001). Water-flow rates and passive diffusion partially explain differential survival of corals during the 1998 bleaching event. Mar. Ecol. Prog. Ser..

[71] Hongo C, Yamano H (2013). Species-specific responses of corals to bleaching events on anthropogenically turbid reefs on Okinawa Island, Japan, over a 15-year Period (1995–2009). PLoS ONE.

[72] Cacciapaglia C, van Woesik R (2016). Climate-change refugia: Shading reef corals by turbidity. Glob. Change Biol..

[73] Wiedenmann J (2013). Nutrient enrichment can increase the susceptibility of reef corals to bleaching. Nat. Clim. Change.

[74] Wooldridge SA, Done TJ (2009). Improved water quality can ameliorate effects of climate change on corals. Ecol. Appl..

[75] Anthony KRN, Kline DI, Diaz-Pulido G, Dove S, Hoegh-Guldberg O (2008). Ocean acidification causes bleaching and productivity loss in coral reef builders. Proc. Natl. Acad. Sci..

[76] Kwiatkowski L, Cox P, Halloran PR, Mumby PJ, Wiltshire AJ (2015). Coral bleaching under unconventional scenarios of climate warming and ocean acidification. Nat. Clim. Change.

[77] Baker AC (2003). Flexibility and specificity in coral-algal symbiosis: Diversity, ecology, and biogeography of *Symbiodinium*. Annu. Rev. Ecol. Evol. Syst..

[78] LaJeunesse TC (2004). High diversity and host specificity observed among symbiotic dinoflagellates in reef coral communities from Hawai’i. Coral Reefs.

[79] Ziegler M (2017). Biogeography and molecular diversity of coral symbionts in the genus *Symbiodinium* around the Arabian Peninsula. J. Biogeogr..

[80] Quigley, K. M., Baker, A. C., Coffroth, M. A., Willis, B. L. & van Oppen, M. J. H. Bleaching resistance and the role of algal endosymbionts in coral bleaching (eds. van Oppen, M. J. H. & Lough, J. M.) vol. 233 111–151 (Springer International Publishing, 2018).

[81] de Souza MR (2022). Community composition of coral-associated Symbiodiniaceae is driven by fine-scale environmental gradients. R. Soc. Open Sci..

[82] Bahr KD, Rodgers KS, Jokiel PL (2017). Impact of three bleaching events on the reef resiliency of Kāne‘ohe Bay, Hawai‘i. Front. Mar. Sci..

[83] Franklin E, Jokiel P, Donahue M (2013). Predictive modeling of coral distribution and abundance in the Hawaiian Islands. Mar. Ecol. Prog. Ser..

[84] Bahr KD, Jokiel PL, Toonen RJ (2015). The unnatural history of Kāne‘ohe Bay: coral reef resilience in the face of centuries of anthropogenic impacts. PeerJ.

[85] Hunter CL, Evans W (1995). Coral reefs in Kāne‘ohe Bay, Hawai‘i two centuries of western influence and two decades of data. Bull. Mar. Sci..

[86] Caruso C (2022). Genetic patterns in *Montipora capitata* across an environmental mosaic in Kāne’ohe Bay. Mol Col..

[87] Lowe RJ, Falter JL, Monismith SG, Atkinson MJ (2009). Wave-driven circulation of a coastal reef–lagoon system. J. Phys. Oceanogr..

[88] Lowe RJ, Falter JL, Monismith SG, Atkinson MJ (2009). A numerical study of circulation in a coastal reef-lagoon system. J. Geophys. Res..

[89] Jury CP, Toonen RJ (2019). Adaptive responses and local stressor mitigation drive coral resilience in warmer, more acidic oceans. Proc. R. Soc. B..

[90] Stat M (2011). Variation in symbiodinium ITS2 sequence assemblages among coral colonies. PLoS ONE.

[91] Innis T, Cunning R, Ritson-Williams R, Wall CB, Gates RD (2018). Coral color and depth drive symbiosis ecology of *Montipora capitata* in Kāne‘ohe Bay, O‘ahu, Hawai‘i. Coral Reefs.

[92] Drury C (2021). Intrapopulation adaptive variance supports selective breeding in a reef-building coral. bioRxiv.

[93] Ritson-Williams R, Gates RD (2020). Coral community resilience to successive years of bleaching in Kāne‘ohe Bay, Hawai‘i. Coral Reefs.

[94] Barott KL (2021). Coral bleaching response is unaltered following acclimatization to reefs with distinct environmental conditions. Proc. Natl. Acad. Sci. USA.

[95] Jones R, Brush E, Dilley E, Hixon M (2021). Autumn coral bleaching in Hawai‘i. Mar. Ecol. Prog. Ser..

[96] Oliver TA, Palumbi SR (2011). Many corals host thermally resistant symbionts in high-temperature habitat. Coral Reefs.

[97] McLachlan RH, Price JT, Solomon SL, Grottoli AG (2020). Thirty years of coral heat-stress experiments: A review of methods. Coral Reefs.

[98] Grottoli AG (2021). Increasing comparability among coral bleaching experiments. Ecol. Appl..

[99] Baker AC (2001). Reef corals bleach to survive change. Nature.

[100] Cunning R, Silverstein RN, Baker AC (2015). Investigating the causes and consequences of symbiont shuffling in a multi-partner reef coral symbiosis under environmental change. Proc. R. Soc. B..

[101] Cunning R, Silverstein RN, Baker AC (2018). Symbiont shuffling linked to differential photochemical dynamics of *Symbiodinium* in three Caribbean reef corals. Coral Reefs.

[102] Thomas L, López EH, Morikawa MK, Palumbi SR (2019). Transcriptomic resilience, symbiont shuffling, and vulnerability to recurrent bleaching in reef-building corals. Mol. Ecol..

[103] Buddemeier RW, Fautin DG (1993). Coral bleaching as an adaptive mechanism. Bioscience.

[104] Møller AP, Jennions MD (2002). How much variance can be explained by ecologists and evolutionary biologists?. Oecologia.

[105] Botté ES (2022). Reef location has a greater impact than coral bleaching severity on the microbiome of *Pocillopora acuta*. Coral Reefs.

[106] Padilla-Gamiño JL, Pochon X, Bird C, Concepcion GT, Gates RD (2012). From Parent to gamete: vertical transmission of *Symbiodinium* (Dinophyceae) ITS2 sequence assemblages in the reef building coral *Montipora capitata*. PLoS ONE.

[107] Barshis DJ (2010). Protein expression and genetic structure of the coral *Porites lobata* in an environmentally extreme Samoan back reef: Does host genotype limit phenotypic plasticity?. Mol. Ecol..

[108] Barshis DJ (2013). Genomic basis for coral resilience to climate change. Proc. Natl. Acad. Sci. USA.

[109] Putnam HM, Edmunds PJ (2011). The physiological response of reef corals to diel fluctuations in seawater temperature. J. Exp. Mar. Biol. Ecol..

[110] Palumbi SR, Barshis DJ, Traylor-Knowles N, Bay RA (2014). Mechanisms of reef coral resistance to future climate change. Science.

[111] Schoepf V, Stat M, Falter JL, McCulloch MT (2015). Limits to the thermal tolerance of corals adapted to a highly fluctuating, naturally extreme temperature environment. Sci. Rep..

[112] Safaie A (2018). High frequency temperature variability reduces the risk of coral bleaching. Nat. Commun..

[113] Ogle K (2015). Quantifying ecological memory in plant and ecosystem processes. Ecol. Lett..

[114] Peterson GD (2002). Contagious disturbance, ecological memory, and the emergence of landscape pattern. Ecosystems.

[115] Hughes TP (2019). Ecological memory modifies the cumulative impact of recurrent climate extremes. Nat. Clim. Change.

[116] Hackerott S, Martell HA, Eirin-Lopez JM (2021). Coral environmental memory: Causes, mechanisms, and consequences for future reefs. Trends Ecol. Evol..

[117] Quigley KM, Willis BL, Kenkel CD (2019). Transgenerational inheritance of shuffled symbiont communities in the coral Montipora digitata. Sci. Rep..

[118] Storlazzi CD, Field ME, Bothner MH (2011). The use (and misuse) of sediment traps in coral reef environments: Theory, observations, and suggested protocols. Coral Reefs.

[119] Guest JR (2016). Coral community response to bleaching on a highly disturbed reef. Sci. Rep..

[120] Jacobs KP (2021). A phylogenomic examination of Palmyra Atoll’s corallimorpharian invader. Coral Reefs.

[121] Martin M (2011). Cutadapt removes adapter sequences from high-throughput sequencing reads. EMBnet.journal.

[122] Hume BCC (2019). SymPortal: A novel analytical framework and platform for coral algal symbiont next-generation sequencing *ITS2* profiling. Mol. Ecol. Resour..

[123] Wyatt ASJ (2020). Heat accumulation on coral reefs mitigated by internal waves. Nat. Geosci..

