# Supplementary material for: Importance of depth and temperature variability as drivers of coral symbiont composition despite a mass bleaching event
Source: Sci Rep. 2023 Jun 2;13:8957. doi: 10.1038/s41598-023-35425-9 (PMC10238383; doi:10.1038/s41598-023-35425-9)
Supplement: Supplementary file 1 — Supplementary Information. [file 41598_2023_35425_MOESM1_ESM.pdf]

Supplemental material

A

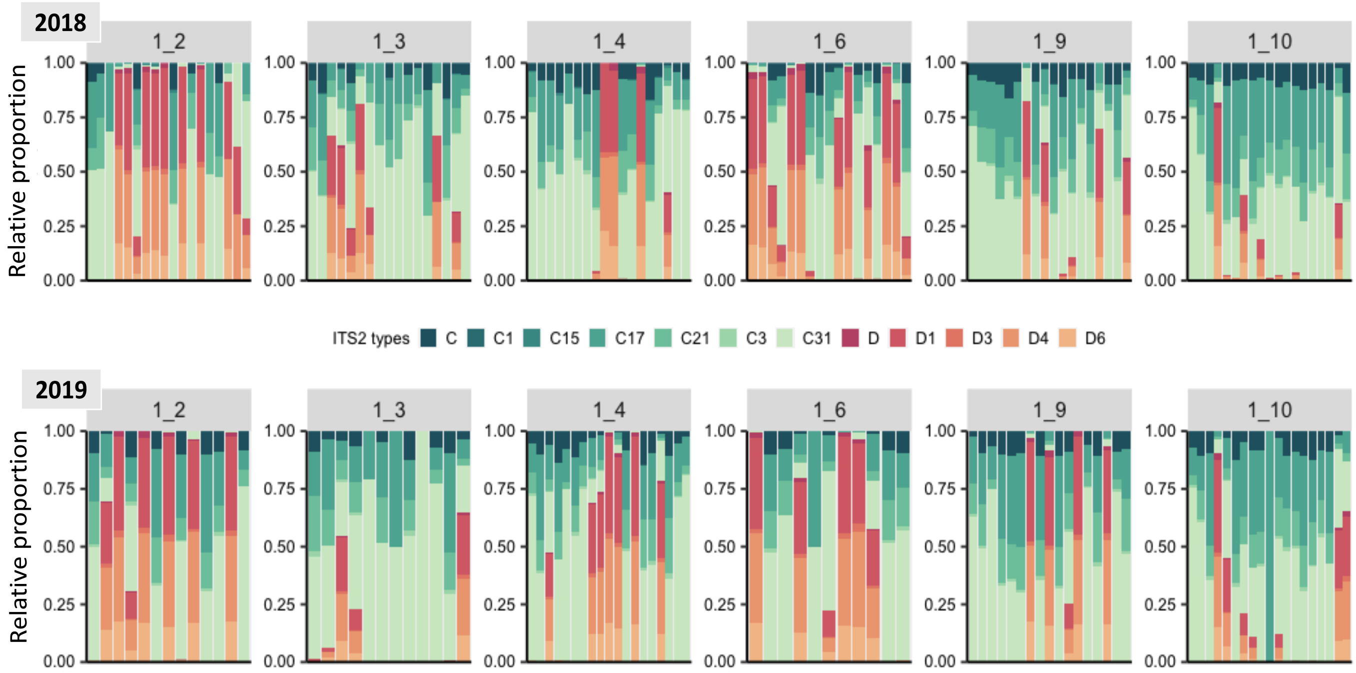

B

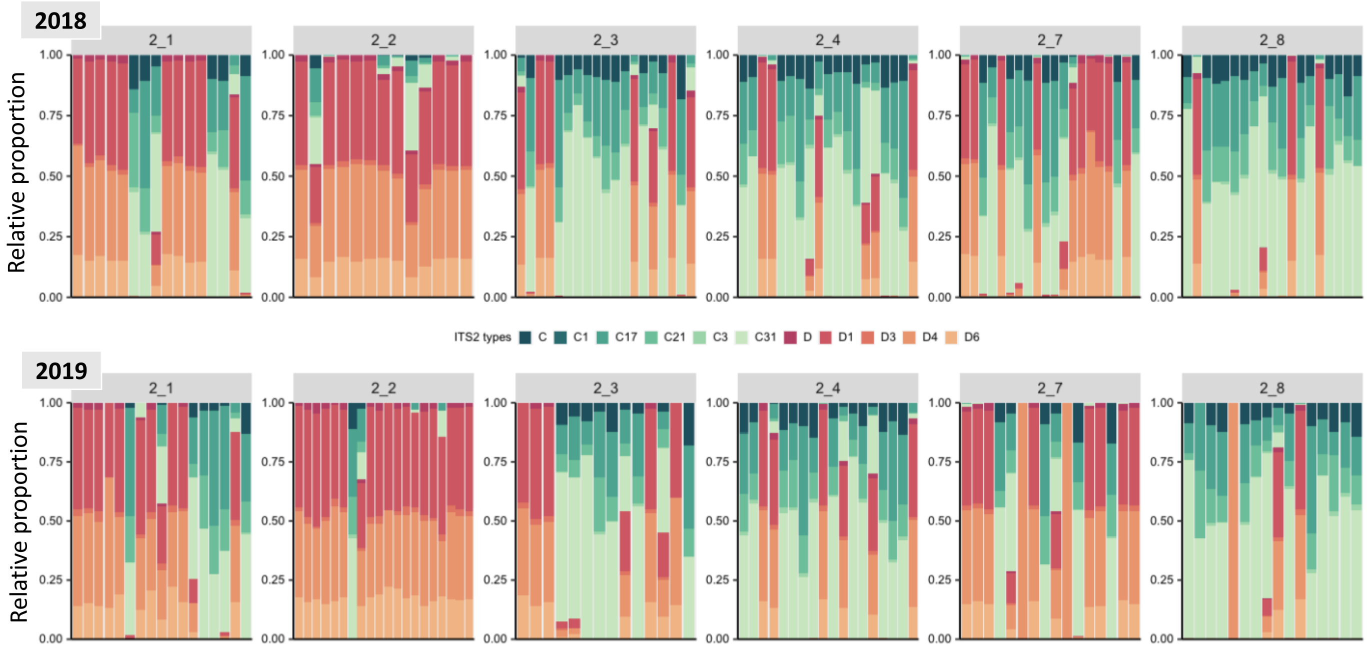

C

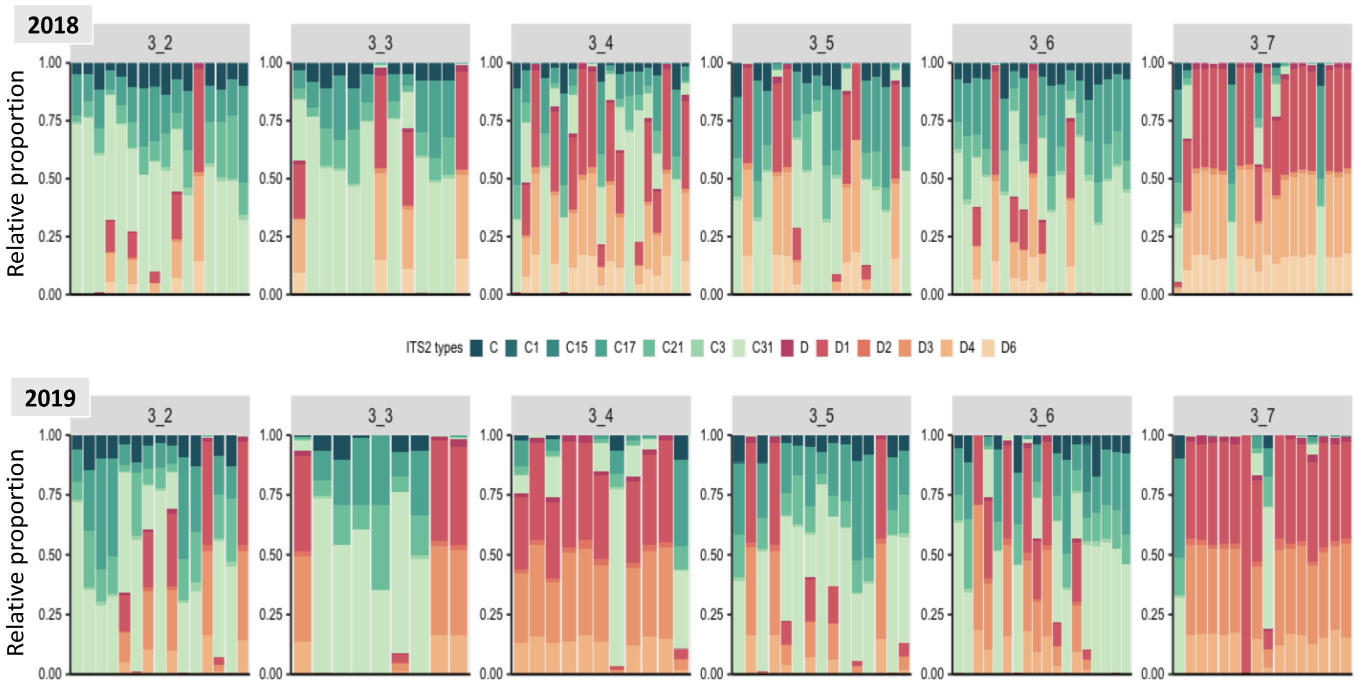
2018
2019
3_2
3_3
3_4
3_5
3_6
3_7
Relative proportion
1.00
0.75
0.50
0.25
0.00
ITS2 types
C
C1
C15
C17
C21
C3
C31
D
D1
D2
D3
D4
D6

D

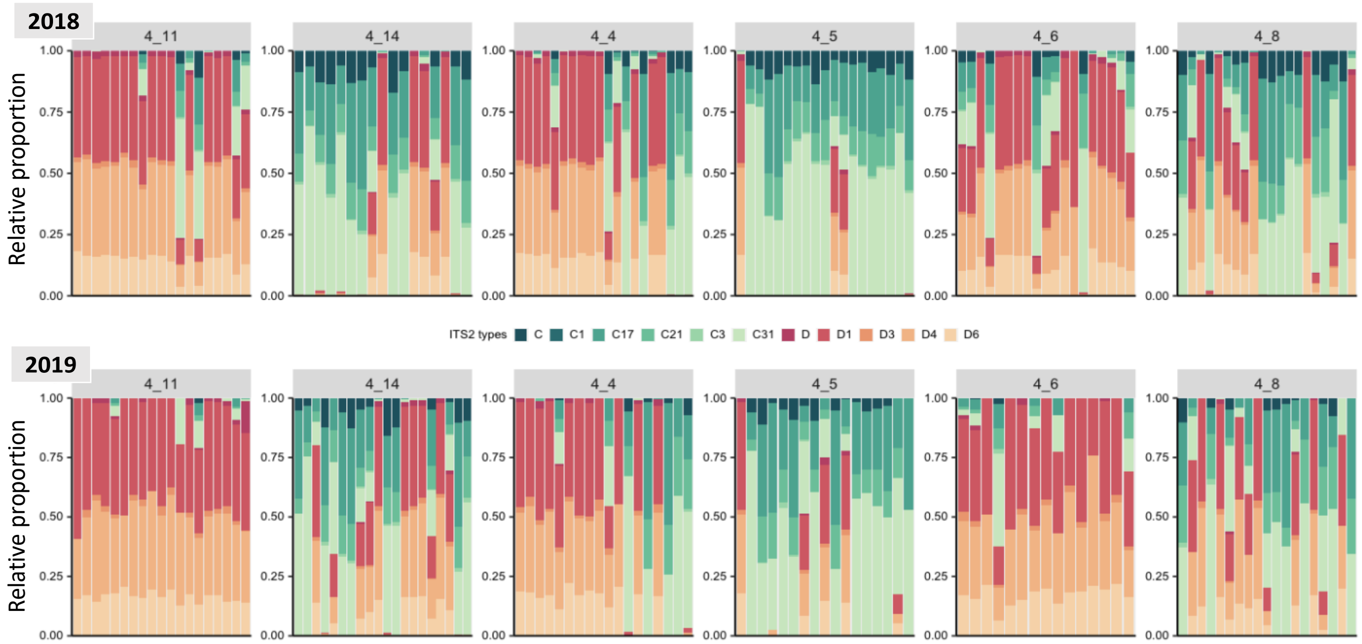
2018
2019
4_11
4_14
4_4
4_5
4_6
4_8
Relative proportion
1.00
0.75
0.50
0.25
0.00
ITS2 types
C
C1
C17
C21
C3
C31
D
D1
D3
D4
D6

E

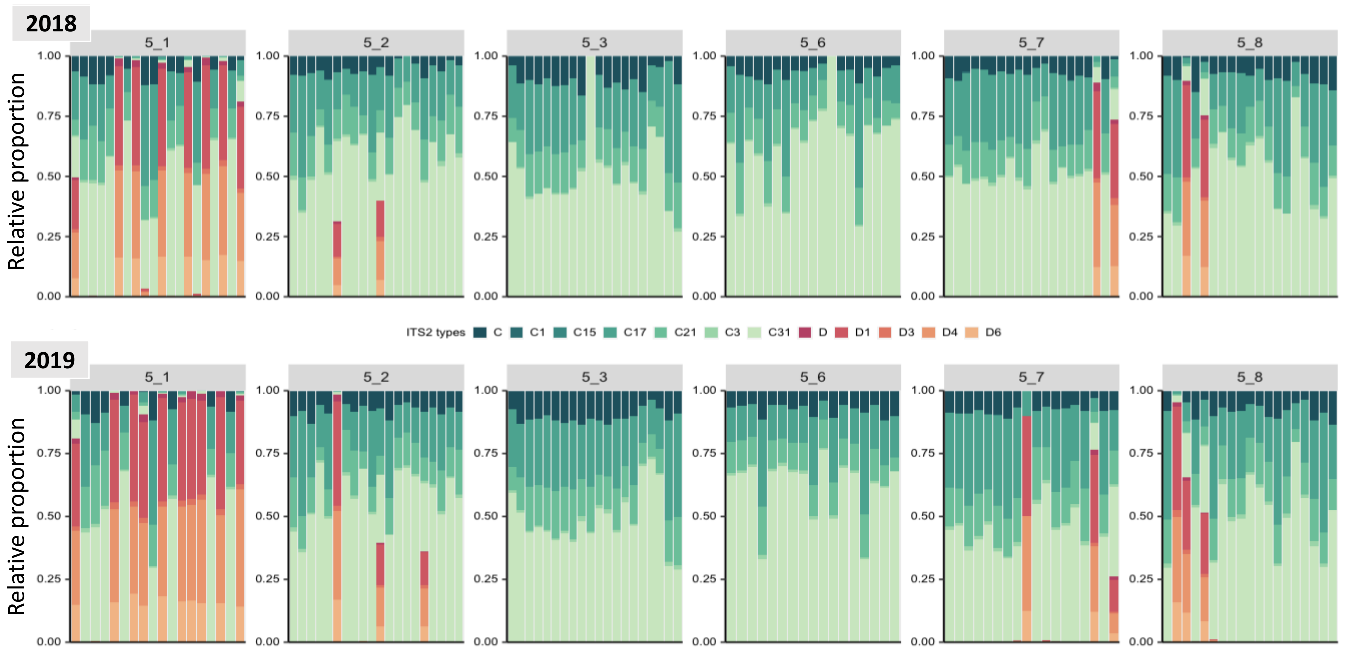

Figure 1. Relative proportion of Symbiodiniaceae types present in each *Montipora capitata* colony in each site in 2018 and 2019. Panels shows the different sites in each of one the blocks - block 1 (A), block 2 (B), block 3 (C), block 4 (D), block 5 (E).

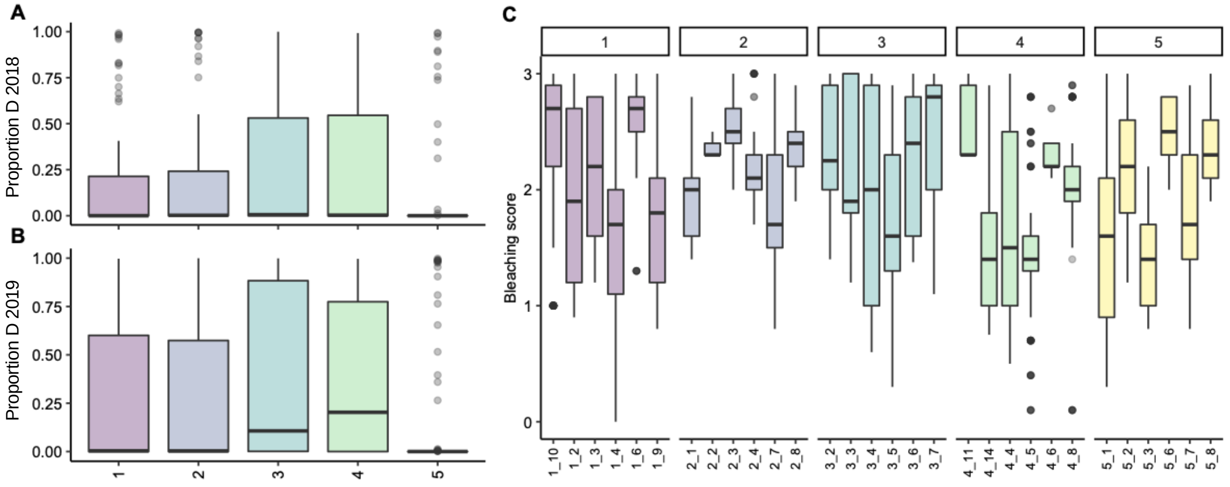

Figure 2. Proportion of *Durusdinium* per block in A) 2018 and B) 2019. C) Bleaching score per site.

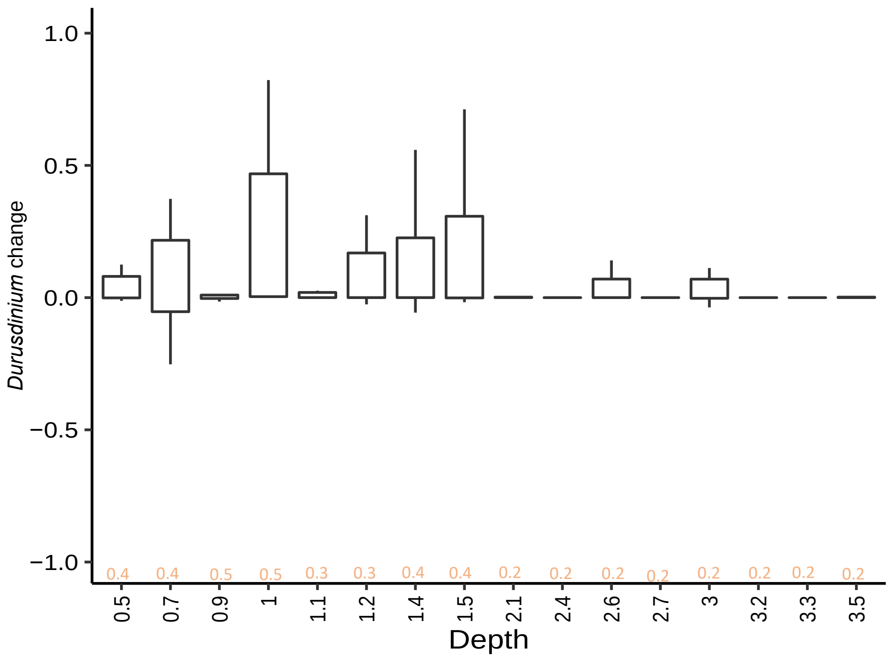

Figure 3. Difference in the proportion of *Durusdinium* (D) in 2019 when compared to the proportion present in 2018 plotted by site depth (in meters). Mean daily temperature standard deviation for each depth are shown above each depth.

Table 1. Site information, sedimentation, and comparison of temperature for 2018 and 2019.

Site	Block	Depth (m)	Sedimentation mean g/day	Temperature max 2018	Temp max 2019
1_2	1	1.2	0.06602	29.3728	29.7687
1_3	1	3	0.02187	28.8250	28.6231
1_4	1	3.5	0.01931	28.8416	29.7490
1_6	1	0.7	0.02287	28.9421	30.2274
1_9	1	2.6	0.02982	28.8125	28.7719
1_10	1	3	0.02928	28.8859	28.6366
2_1	2	1.2	0.16410	28.8539	29.4408
2_2	2	0.5	0.04501	28.8794	28.7067
2_3	2	0.5	0.02779	29.2753	30.1186
2_4	2	2.4	2.93056	28.6209	29.0228
2_7	2	1.5	0.40130	28.7231	30.5101
2_8	2	2.1	0.27611	28.7231	28.9946

3_2	3	1.4	0.09804	28.1498	28.9693
3_3	3	1	0.05737	28.9506	28.6542
3_4	3	1	0.04163	29.2845	29.4586
3_5	3	0.9	0.03061	29.0320	30.1222
3_6	3	0.9	0.03796	28.9826	31.9997
3_7	3	0.7	0.02244	29.1680	29.6930
4_4	4	1.4	0.04811	28.9492	30.0067
4_5	4	1.1	0.03305	28.9298	29.6440
4_6	4	1.5	0.34257	29.1865	31.1699
4_8	4	1.2	0.01573	28.8363	29.9775
4_11	4	1.4	0.07752	29.2206	30.1610
4_14	4	1.4	0.16850	28.9260	30.0172
5_1	5	1.2	0.21580	29.3822	29.7978
5_2	5	2.7	0.62898	28.8171	28.5985
5_3	5	2.4	0.13268	28.7982	28.5721
5_6	5	3.5	0.41842	27.6119	30.0287
5_7	5	3.3	0.38405	28.6961	29.6721
5_8	5	3.2	0.28655	28.7486	29.6730

Table 2. Summary statistics per block in 2018 and 2019

Block	Mean ºC 2018	Mean ºC 2019	Max ºC 2018	Max ºC 2019	Daily ºC range 2018	Daily ºC range 2019	Daily ºC standard deviation 2018	Daily ºC Standard deviation 2019
**1**	25.697	26.207	28.946	29.296	0.244	1.007	0.243	0.264
**2**	25.647	26.265	28.834	29.465	1.246	1.333	0.335	0.355
**3**	25.798	26.335	28.928	29.816	1.871	2.014	0.474	0.505
**4**	25.716	26.536	29.008	30.162	1.261	1.383	0.342	0.380
**5**	25.490	26.159	28.675	29.390	0.746	0.756	0.204	0.212
